# High FOXP3+ regulatory T-cell density in the sentinel lymph node is associated with downstream non-sentinel lymph-node metastasis in gastric cancer

**DOI:** 10.1038/bjc.2011.248

**Published:** 2011-07-05

**Authors:** H E Lee, D J Park, W H Kim, H H Kim, H S Lee

**Affiliations:** 1 Department of Pathology, Seoul National University Hospital, 28 Yeongon-dong, Jongno-gu, Seoul 110-744, Korea; 2 Department of Surgery, Seoul National University Bundang Hospital, 166 Gumi-ro, Bundang-gu, Seongnam-si, Gyeonggi 463-707, Korea; 3 Department of Surgery, Seoul National University College of Medicine, 28 Yeongon-dong, Jongno-gu, Seoul 110-799, Korea; 4 Department of Pathology, Seoul National University Bundang Hospital, 166 Gumi-ro, Bundang-gu, Seongnam-si, Gyeonggi 463-707, Korea

**Keywords:** stomach neoplasms, sentinel lymph nodes, regulatory T cells, dendritic cells, tumour immunology

## Abstract

**Background:**

We aimed to evaluate the immunologic nature of sentinel lymph nodes (SLNs) in gastric cancer patients and to determine whether it can predict non-SLN metastasis.

**Methods:**

Sentinel lymph node samples were collected from 64 gastric carcinoma patients who had undergone gastrectomy with SLN biopsy. One representative SLN sample was selected from each patient and was subjected to immunostaining for CD8, CD57, FOXP3, and DC-LAMP. The numbers of marker-positive cells in each sample were counted. The relationships between various immune cell densities and clinicopathologic parameters or metastasis status of SLNs and non-SLNs were sought.

**Results:**

High FOXP3+ Treg density of the SLN was found to be significantly associated with the presence of metastasis in either SLNs or non-SLNs. DC-LAMP+ cell density of the SLN was the highest at the isolated tumours cell level, and this decreased along with an increase in tumour metastasis in either SLNs or non-SLNs. Univariate and multivariate logistic regression models revealed that high FOXP3+ Treg density of the SLN was an independently significant predictor of non-SLN metastasis.

**Conclusions:**

This study is the first to indicate an important role of SLNs in metastatic dissemination of gastric cancer. Our findings suggest that Tregs could be a new therapeutic target for regulating the metastasis of gastric cancer.

In the treatment of gastric cancer, the standard procedure has been complete excision of both the primary tumour and the regional lymph nodes (LNs). However, metastasis to regional LNs is observed in only 2–8% and 40–50% of pT1 and pT2 gastric cancer patients, respectively (Schlag *et al*, 2004; Bembenek *et al*, 2007); and thus, unnecessary LN dissection is performed in >50% of pT1-2 patients. In addition, prophylactic LN dissection exposes patients to various complications that result in increased mortality and morbidity. Given this background, the sentinel LN (SLN) concept with intraoperative lymphatic mapping and SLN biopsy has emerged recently.

The SLN is defined as the LN first receiving lymphatic drainage from the site of the primary tumour. Since the SLN concept was first advocated by Morton *et al* (1992) in patients with malignant melanoma, SLN mapping and biopsy have become widely accepted in routine surgical management of malignant melanoma and breast cancers (Moroi, 2009; Quan and McCready, 2009). Recently, the SLN concept was also introduced to gastric cancer, and there have been many studies on the feasibility and accuracy of SLN mapping and biopsy (Arigami *et al*, 2006; Saikawa *et al*, 2006; Lee *et al*, 2008, 2009; Takeuchi *et al*, 2008, 2010; Ichikura *et al*, 2009). However, the clinical application of SLN navigation surgery on gastric cancer is still under discussion because of the various results of sensitivities and false negatives in SLN-based diagnosis. In addition, the frequency of skip metastasis has been reported to be much higher in gastric cancer than in breast cancer or melanoma, possibly because of the complex embryologic development and lymphatic drainage of the stomach (Schlag *et al*, 2004; Tangoku *et al*, 2007). However, if it is possible to predict the risk of non-SLN metastasis accurately before or during the operation and thus not overlook cases with skip metastasis, SLN navigation surgery could become acceptable as a safe procedure for gastric cancer management. In addition, the accurate prediction of non-SLN metastasis would enable us to identify patients who could avoid potential morbidity associated with the more radical procedure.

The SLN is a preferential site for initial metastasis of solid tumours but is also regarded as the first lymphoid organ to respond to tumour antigen stimulation (Takeuchi *et al*, 2008). Within the SLN, immunoreactive lymphocytes initially encounter tumour-specific antigens and develop antitumour immunity. In brief, antigen-acquiring dendritic cells (DCs) pass from the site of the primary tumour to the regional LN, wherein they present antigens to naive T lymphocytes, converting them into specifically sensitised T lymphocytes such as helper T lymphocytes or cytotoxic T lymphocytes. Subsequently, some of them circulated back to the primary tumour where they display an antitumour effect (Huang *et al*, 2000). On the basis of our knowledge, immune responses in SLNs are expected to have an important role in preventing further spread of the tumour; and therefore, investigation of the immunologic status of SLNs, including DC maturation and T-cell responses, is one of the most significant issues in tumour immunology.

Previous studies demonstrated that the immunoreactivity of SLNs is entirely or segmentally downregulated as compared with that of non-SLNs in breast cancer and melanoma, as evidenced by a reduction in the frequency, density, meshworking, and dendritic complexity of DCs and by a reduction in the density and activation marker expression of T cells in the paracortex (Shu *et al*, 2006). The immune suppression of the SLN is considered to be induced by immunomodulators produced by primary tumour cells (Shu *et al*, 2006). In addition, previous reports indicated that the immune profile in metastasis-free SLNs is different from that in metastasis-involved SLNs in breast cancer and melanoma, although they showed contradictory results (Movassagh *et al*, 2004; Poindexter *et al*, 2004; Matsuura *et al*, 2006, 2009; Mansfield *et al*, 2009; Nakamura *et al*, 2009). However, immunologic roles of SLNs in the development of tumour metastasis have not yet been completely elucidated. Moreover, there has been only one report that has addressed the immunologic status of SLNs in gastric cancer (Ishigami *et al*, 2003), and furthermore, there are no reports that compare the immune status or the SLN with the metastasis status of either SLNs or non-SLNs.

The aim of this study was to evaluate the immunologic nature of the SLN in gastric cancer and to determine whether it can predict downstream non-SLN metastasis. Here, we investigated the density of CD8+, FOXP3+, CD57+, and DC-LAMP+ immune cells in the SLN and found that the density of FOXP3+ regulatory T cell (Treg) is an independent predictive factor of non-SLN metastasis in gastric cancer.

## Patients and methods

### Patients

We collected SLN samples from 64 patients (35 men and 29 women) with cT1-2, NO gastric cancer, for whom SLN mapping and biopsy and subsequent laparoscopic gastrectomy were performed at Seoul National University Bundang Hospital. No patient had received preoperative chemotherapy or radiotherapy. The pathologic tumour-node-metastasis stage (according to AJCC seventh edition) and histologic classification (according to the World Health Organization) were evaluated after the operation. Histologic findings showed that 35 patients (54.7%) had pT1a cancer; 19 (29.7%), pT1b cancer; 5 (7.8%), pT2 cancer; 4 (6.3%), pT3 cancer; and 1 (1.6%), pT4a cancer. There were 31 (48.4%) and 33 (51.6%) cases that were classified as intestinal and diffuse type of Lauren classification, respectively. The tumour was located in the lower third of the stomach in 35 cases (54.7%) and in the middle third in 29 cases (45.3%). This study was approved by the Institutional Review Board for research using human subjects at Seoul National University Bundang Hospital.

### Determination of metastasis status of SLNs and non-SLNs

We previously determined the metastasis status of SLNs and non-SLNs with the following pathologic examination method (under submission). Briefly, 207 SLN samples from 64 patients were examined. During the operation, we sliced the samples at 2 mm intervals in the plane of the largest dimension. All pieces were frozen, sectioned, and stained with haematoxylin and eosin (HE) (two serial cuts; thickness, 4 *μ*m). In 30 cases, an additional serial cut was stained for rapid immunohistochemistry (IHC) using an SLN rapid IHC kit (28-8700; Zymed Laboratories, South San Francisco, CA, USA). The SLN portions remaining after intraoperative examination were formalin fixed and paraffin embedded. All dissected non-SLNs were sliced and stained with HE. Two serial slides were obtained and stained with HE and subjected to immunostaining for CK (1 : 300; monoclonal antibody anti-human cytokeratin clone MNF116; Dako, Glostrup, Denmark) using an automated immunostainer (BenchMark XT; Ventana Medical Systems Inc., Tucson, AZ, USA) according to the manufacturer’s instructions. Nodal metastases were subdivided into three groups according to AJCC guidelines using the maximum size of tumour deposits: isolated tumours cells (ITCs), micrometastases, and macrometastases. Finally, 45 (70.3%), 4 (6.3%), 11 (17.2%), and 4 (6.3%) cases showed negativity, ITCs, micrometastases, and macrometastases in SLNs, respectively; and 49 (76.6%), 3 (4.7%), 4 (6.3%), and 8 (12.5%) showed negativity, ITCs, micrometastases, and macrometastases in non-SLNs, respectively. When only micrometastasis and macrometastasis were considered as presence of metastasis, two cases showed skip metastasis, which represent non-SLN metastasis without any SLN metastasis, and five cases displayed SLN metastasis but no further metastasis in non-SLNs.

### Immunohistochemical staining for CD8, CD57, FOXP3, and DC-LAMP

Formalin-fixed, paraffin-embedded SLNs were sectioned into 4 *μ*m slices and affixed onto glass slides. Sections were obtained from a representative SLN sample from each patient and were subjected to immunostaining for CD8 (cytotoxic T-cell marker; 1 : 50; 1A5; Dinona, Seoul, Korea), FOXP3 (Treg marker; 1 : 100; 236A/E7; Abcam, Cambridge, UK), CD57 (natural killer (NK) cell marker; 1 : 150; NK-1; Zymed), and DC-LAMP (mature DC marker; 1 : 50; 104.G4; Beckman Coulter, Brea, CA, USA) using an automated immunostainer (BenchMark XT, Ventana Medical Systems Inc.) according to the manufacturer’s instructions.

### Quantitative analysis of immunostaining-positive cells

Slides subjected to immunostaining were analysed using NIH ImageJ software with a Java-based colour deconvolution plugin (http://rsb.info.nih.gov/ij). The numbers of antibody-positive immune cells in each case were counted in two representative areas at a magnification of × 100. The mean numbers of immune cells in two different areas were calculated, and their density was determined for each patient.

### Statistical analyses

Continuous variables were compared using the Mann–Whitney *U*-test or Kruskal–Wallis test. Univariate and multivariate logistic regression models were used to identify predictive factors of non-SLN metastasis. The covariates, which were statistically significant in the univariate analysis, were then included in the multivariate analysis; the odds ratio with its 95% confidence interval (CI) was assessed for each factor. All statistical analyses were conducted using SPSS statistics 17.0 (SPSS Inc., Chicago, IL, USA), and *P*-values ⩽0.05 were considered statistically significant.

## Results

### Density of various immune cells in SLNs

**Figure 1:**
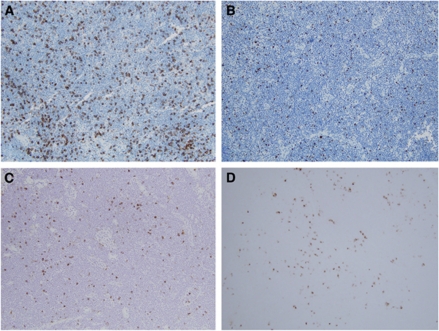
Immunohistochemical staining for various types of immune cells in the SLN from gastric cancer patients. Density of CD8+ cytotoxic T cells (**A**), FOXP3+ regulatory T cell (**B**), CD57+ NK cells (**C**), and DC-LAMP+ mature DCs (**D**) was determined (original magnifications, × 100).

We identified CD8+, FOXP3+, CD57+, and DC-LAMP+ immune cells in SLNs using IHC (Figure 1). CD8+ cells were mostly in the paracortex. In the cases of FOXP3+ cells, the majority resided in the paracortex, but a small number of them were scattered in the follicles. CD57+ cells were found not only in the paracortex but also in the germinal centres of the follicles. Most DC-LAMP+ cells were presented surrounding the paracortex. The distribution pattern of these cells did not differ according to the metastasis status of SLNs.

The median numbers of CD8+, FOXP3+, CD57+, and DC-LAMP+ immune cells of SLNs were 2686 (range, 870–4715), 928.5 (42–2347), 592 (8–1371), and 377 (46–1040), respectively. Correlations between the numbers of the various immune cells were not statistically significant, except for the numbers of CD8+ and FOXP3+ immune cells, which were significantly correlated (correlation coefficient, 0.310; *P*=0.015).

### Correlation between various immune cell densities of the SLN and clinicopathologic characteristics

Among 64 gastric cancer patients, the median age was 57.5 years (range, 31–78 years), and 54.7% were men. Associations between immune cell density of SLNs and clinicopathologic characteristics are summarised in Table 1. Diffuse histologic-type cancers tended to have more CD8+, FOXP3+, CD57+, and DC-LAMP+ immune cells than intestinal-type cancers. Although no significant association was observed between the invasion depth of the primary tumour and densities of various immune cells, the SLN of pT2-4 cancers showed a relatively high FOXP3+ T-cell density compared with that of pT1 cancers. In addition, high FOXP3+ T-cell density was significantly related to regional LN metastasis.

### Correlation between various immune cell densities of the SLN and metastasis status of SLNs

**Figure 2:**
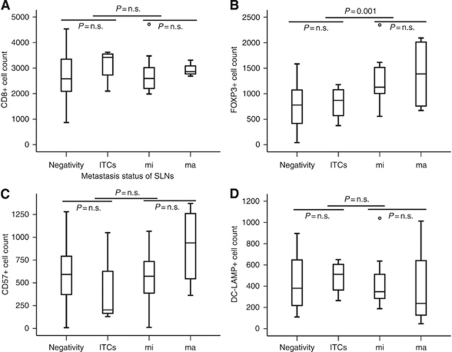
Association between various immune cell densities of the SLN and metastasis status of SLNs in gastric cancer. Median values with interquartile ranges of CD8+ (**A**), FOXP3+ (**B**), CD57+ (**C**), and DC-LAMP+ (**D**) cell densities are represented according to metastasis status of SLNs in gastric cancer. *P,* Mann-Whitney *U*-test. Numbers of cases with negativity, isolated tumour cells (ITCs), micrometastasis (mi), and macrometastasis (ma) in SLNs are 45, 4, 11, and 4, respectively.

FOXP3+ T-cell density of the SLN tended to increase along with enlargement of tumour metastasis in SLNs; the difference in density within SLNs was statistically significant when comparing the cases having micrometastases or macrometastases in SLNs with those showing ITCs or negativity (*P*=0.001). DC-LAMP+ cell density of the SLN was highest at the ITC level and was found to decrease as tumour metastasis in SLNs increased. CD8+ or CD57+ cell density of the SLN was not associated with the metastasis status of SLNs (Figure 2).

### Correlation between various immune cell densities of the SLN and metastasis status of non-SLNs

**Figure 3:**
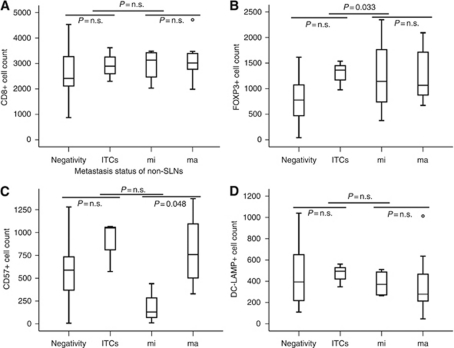
Association between various immune cell densities of the SLN and metastasis status of non-SLNs in gastric cancer. Median values with interquartile ranges of CD8+ (**A**), FOXP3+ (**B**), CD57+ (**C**), and DC-LAMP+ (**D**) cell densities are represented according to metastasis status of non-SLNs in gastric cancer. *P,* Mann–Whitney *U*-test. Numbers of cases with negativity, isolated tumour cells (ITCs), micrometastasis (mi), and macrometastasis (ma) in non-SLNs are 49, 3, 4, and 8, respectively.

Analyses of relationships between the immunologic status of SLNs and non-SLN metastasis revealed that high FOXP3+ T-cell density of the SLN was significantly associated with the presence of non-SLN metastasis (*P*=0.033). Similar to the comparison with metastasis status of SLNs, DC-LAMP+ cell density of the SLN was highest at the ITC level, and was found to decrease as tumour metastasis in non-SLNs increased. In non-SLNs, CD57+ cell density of the SLN was significantly higher in the cases of macrometastases than in those of micrometastases (*P*=0.048). No significant association was found between CD8+ cell density of the SLN and metastasis status of non-SLNs (Figure 3).

### Immune cell density of SLNs as a predictor of non-SLN metastasis

A logistic regression model was used to evaluate the relative power of the various factors to predict the likelihood of metastatic gastric carcinoma in downstream non-SLNs. We performed the analysis with clinicopathologic factors, which could be obtained before the operation, and the immune status of the SLN. FOXP3+ T-cell density of the SLN, primary tumour size, and Lauren classification were selected as significant predictors of non-SLN metastasis, including micrometastases and macrometastases, by this approach. In multivariate analysis, FOXP3+ T-cell density of the SLN remained an independent predictor of non-SLN metastasis with odds ratio (95% CI) of 1.002 (1.000–1.003) (Table 2).

## Discussion

Because the SLN is the site where naive lymphocytes initially encounter tumour-specific antigens and develop antitumour immunity (Takeuchi *et al*, 2008), it has been expected that the immune status of SLNs would have a considerable effect on the metastatic spread of cancer. In the present study, we preferentially described the immunologic nature of the SLN by determining the density of CD8+, FOXP3+, CD57+, or DC-LAMP+ immune cells in gastric cancer. Next, we investigated the association between the immune status of the SLN and metastasis status of either SLNs or non-SLNs, and revealed that high Treg density of the SLN is related to the presence of metastasis in either SLNs or non-SLNs. Furthermore, it was found that high Treg density in the SLN is an independent predictor of downstream non-SLN metastasis. This study is the first to reveal the associations between the immune status of the SLN and metastasis status of SLNs or non-SLNs. Thus far, there has been only one study describing the immune profiles of SLNs, in which the comparisons of the immune cell density were performed between SLNs and non-SLNs (Ishigami *et al*, 2003), although a few previous reports investigated immune status of regional LNs obtained from gastric cancer patients who did not undertake the SLN navigaion surgery (Ishigami *et al*, 2000; Nagata *et al*, 2004; Kawaida *et al*, 2005; Maruyama *et al*, 2010).

The techniques of lymphatic mapping and SLN biopsy have been accepted with great enthusiasm since their first introduction in 1992. The attraction of the approaches is in their ability to identify before the operation the lymph node most likely to contain an early metastatic tumour. It has been known that the presence or absence of a tumour in the SLN is quite a good predictor of whether tumours are present in non-SLNs in melanoma or breast cancer (Cochran *et al*, 2004; Quan and McCready, 2009). In the cases of gastric cancer, however, the predictive power of SLN metastasis status on non-SLN metastasis is relatively low; this may be attributable to the high frequency of skip metastasis due to complex embryologic development and lymphatic drainage of the stomach (Schlag *et al*, 2004; Tangoku *et al*, 2007). Hence, there has been an urgent need to identify predictive factors of downstream non-SLN metastasis in gastric cancer, other than SLN metastasis status. In this study, we tried to identify predictors of non-SLN metastasis among parameters that can be obtained before or during the operation, and found that a high Treg density of the SLN, large tumour size (⩾3 cm), and diffuse histologic-type of primary tumour are predictive factors, identified by univariate logistic regression. Of these, high Treg density is an independent predictor confirmed by multivariate analysis. This study is the first to identify predictive factors of non-SLNs metastasis using logistic regression models with clinicopathologic parameters and immune profiles of the SLN in gastric cancer. However, for application in a clinical setting, more precise predictive models should be established through various analyses involving more factors and a larger population.

FOXP3+ Tregs have a pivotal role in maintaining immune system homeostasis through their ability to suppress immunologic responses, including tumour immunity against tumour-associated antigens (Mansfield *et al*, 2009; Matsuura *et al*, 2009; Nakamura *et al*, 2009). In some previous reports investigating Tregs in SLNs from breast cancer patients, Treg density in the SLN was higher in the tumour-involved SLN, including the node having ITCs or only molecularly detected tumour cells, than in the tumour-free SLN, and these results are concordant with our findings (Mansfield *et al*, 2009; Matsuura *et al*, 2009; Nakamura *et al*, 2009). In the present study, high Treg density of the SLN was associated with the presence of non-SLN metastasis as well. These findings indicate that Tregs of SLNs suppress the antitumour immune response within SLNs, and eventually promote metastatic dissemination of tumours in gastric cancer. On the other hand, one previous report on colorectal cancer showed that low Treg density in negative SLNs was associated with node metastases, which was in contrast to our observations, and suggested that Treg expression correlated with increased tumour protection and survival and was indicative of a successful immune response (Matera *et al*, 2010). To resolve this issue, more data are required on this subject.

Central to the induction of cellular immune responses to antigens are professional antigen-presenting cells, out of which mature DCs are the principal initiators of antigen-specific immune responses followed by the antitumour activity of T cells (mainly cytotoxic T cells) (Shu *et al*, 2006). Some previous reports found that tumour-involved SLNs contained more mature DCs than tumour-free SLNs in breast cancer (Poindexter *et al*, 2004; Matsuura *et al*, 2006, 2009). In particular, it was proposed that the immune status of SLNs, including DC maturation and Th1 responses, was depressed before metastasis, but upregulated after the establishment of metastasis; this was followed by the downregulation of Th1 responses along with an increase in tumour metastasis in SLNs (Matsuura *et al*, 2009). Consistent with the proposal, the present study showed that the mature DC density, which was relatively low in tumour-free SLNs, was highest at the ITC level but decreased along with an increase in tumour metastasis in SLNs. Another previous report demonstrated a significant correlation between mature DC density of SLNs and the absence of metastasis in downstream LNs in melanoma (Movassagh *et al*, 2004). We also showed that mature DC infiltrates tended to be few in the cases of micrometastases or macrometastases in non-SLNs compared with the cases of negativity or ITCs, although the difference was not statistically significant. However, we could not find any significant association between the density of cytotoxic T cells, which are the main effectors of antitumour immunity, in the SLN and metastasis status of SLNs or non-SLNs. Further studies will be required to elucidate the role of cytotoxic T cells within SLNs in the metastatic spread of tumours.

Previously, it was found that NK cell-mediated immunity has an important role in the inhibition of LN metastasis, especially in the early stage of metastasis development, and that the main factor responsible for the regression of ITCs after primary tumour resection is the immunologic activity conferred by NK cells against metastatic tumour cells in the LNs (Yokoyama *et al*, 2006). There were only a few previous studies on NK cells of LNs obtained from gastric cancer patients (Ishigami *et al*, 2000, 2003), and one study reported that a high level of intranodal NK cell infiltration was associated with the absence of disease recurrence in gastric cancer, emphasising the antitumour effect of NK cells within LNs (Ishigami *et al*, 2000). We tried to demonstrate the antitumour activity of NK cells by investigating whether there is an association between NK cell density in the SLN and metastasis status of SLNs or non-SLNs; however, we could not find any consequential findings. The negative result can be attributable to the relatively low number of cases; and therefore, large-scale studies may help ascertain this assumption.

Significant advances have been made in the field of cancer immunotherapy over the last decade, some of which was based on immunomodulation in SLNs to prevent or eradicate tumour metastasis in SLNs (Vuylsteke *et al*, 2004; Kuwashima *et al*, 2005; Salcedo *et al*, 2006). Specifically, among the candidate molecules of immunotherapy, granulocyte-macrophage colony-stimulating factor, interleukin-13, and interferon-*α* act as immunomodulators of SLNs (Vuylsteke *et al*, 2004; Kuwashima *et al*, 2005; Salcedo *et al*, 2006). In order to make the immunotherapy based on SLN activation successful and to develop the novel candidates of immunotherapy as well, the correct identification of the immunologic nature in SLNs may be crucial. Furthermore, our findings suggest that Tregs could be a new therapeutic target for regulating the metastatic spread of gastric cancer.

In conclusion, we revealed that high Treg density of the SLN is associated with SLN or non-SLN metastasis and, through logistic regression models, is an independent predictor of downstream non-SLN metastasis in gastric cancer. We believe that the findings can help decide the extent of gastrectomy and lymphatic dissection to be performed, and thus offer a more individualised therapy for patients in the near future. In addition, our findings suggest that Tregs within the SLN have an important role in promoting metastatic dissemination of cancer. We expect Tregs to be a new therapeutic target for regulating the metastasis of gastric cancer.

## Figures and Tables

**Table 1:** Associations between various immune cell densities of the SLN and clinicopathologic parameters of the primary tumour

		**CD8 (median (IQR))**	**FOXP3 (median (IQR))**	**CD57 (median (IQR))**	**DC-LAMP (median (IQR))**
Sex (no.)	Male (35)	2756 (2242–3343)	843 (496–1313)	596 (257–944)	403 (220–652)
	Female (29)	2339 (2058–3480)	974 (478–1167)	592 (512–700)	365 (220–561)
Age (no.)	⩽65 years (49)	2665 (2131–3323)	843 (477–1203)	603 (350–900)	381 (218–632)
	>65 years (15)	2711 (2128–3661)	1042 (763–1313)	590 (352–676)	372 (266–659)
Lauren (no.)	Intestinal (31)	2643 (2093–3268)	843 (483–1237)	562 (302–670)^*^	377 (211–643)
	Diffuse (33)	2871 (2220–3480)	1001 (518–1296)	676 (502–1038)^*^	388 (266–644)
Tumour size (no.)	<3 cm	2871 (2373–3456)	961 (507–1186)	588 (333–901)	328 (207–583)
	⩾3 cm	2298 (1984–3310)	878 (438–1389)	644 (368–791)	450 (269–653)
pT stage (no.)	T1 (35)	2825 (2279–3418)	778 (418–1229)	584 (278–689)	372 (212–647)
	T2–4 (29)	2515 (2030–3310)	1001 (614–1308)	678 (363–935)	381 (233–636)
LN metastasis (no.)	No (47)	2579 (2088–3418)	778 (418–1074)^**^	592 (369–803)	394 (218–650)
	Yes (17)	2892 (2221–3360)	1127 (876–1576)^**^	608 (343–976)	297 (243–512)
LI (no.)	No (57)	2659 (2128–3405)	949 (477–1218)	591 (370–792)	381 (227–642)
	Yes (7)	2772 (2172–3578)	908 (843–1933)	727 (291–1150)	267 (209–660)

Abbreviations: IQR=interquartile range (25–75%); LI=lymphatic invasion; LN=lymph node; pT=invasion depth of the primary tumour; SLN=sentinel lymph node.

Mann–Whitney *U*-test was used in each analysis; ^*^P<0.05; ^**^P<0.01.

**Table 2:** Univariate and multivariate logistic regression models for predictors of downstream non-SLN metastasis

	**Univariate analysis^a^**			**Multivariate analysis^a^**		
**Variables**	**Odds ratio**	**95% CI**	***P*-value**	**Odds ratio**	**95% CI**	***P*-value**
CD8 count (continuous)	1.001	1.000–1.001	0.135	—	—	—
CD57 count (continuous)	1.000	0.998	1.002	—	—	—
FOXP3 count (continuous)	1.002	1.000–1.003	0.013	1.002	1.000–1.003	0.035
DC-LAMP count (continuous)	0.999	0.996–1.001	0.352	—	—	—
Age (<65 years *vs* ⩾65 years)	0.000	0.000	0.998	—	—	—
Sex (male *vs* female)	0.540	0.145–2.017	0.359	—	—	—
Tumour size (<3 cm *vs* ⩾3 cm)	6.818	1.356–34.274	0.020	4.994	0.898–27.767	0.066
Lauren (intestinal *vs* diffuse)	6.304	1.255–31.660	0.025	5.613	0.946–33.286	0.058

Abbreviations: CI=confidence interval; SLN=sentinel lymph node.

a Univariate or multivariate logistic regression was used.
